# Supplementary material for: Mechanisms governing the pioneering and redistribution capabilities of the non-classical pioneer PU.1
Source: Nat Commun. 2020 Jan 21;11:402. doi: 10.1038/s41467-019-13960-2 (PMC6972792; doi:10.1038/s41467-019-13960-2)
Supplement: Supplementary file 7 — Source data [file 41467_2019_13960_MOESM7_ESM.zip › Source_Data/FigureS2/FigureS2B_MotifScanOutput/KmeansCluster14/homerResults/motif6.similar.html]

motif6

## Information for motif6

A
G
C
T
C
G
A
T
A
C
G
T
A
G
T
C
T
G
A
C
A
C
G
T
A
T
C
G
A
C
G
T
  
Reverse Opposite:  

C
G
T
A
A
T
G
C
C
G
T
A
A
C
T
G
A
C
T
G
G
T
C
A
C
G
T
A
T
C
G
A
  

|  |  |
| --- | --- |
| p-value: | 1e-27 |
| log p-value: | -6.327e+01 |
| Information Content per bp: | 1.866 |
| Number of Target Sequences with motif | 113.0 |
| Percentage of Target Sequences with motif | 22.16% |
| Number of Background Sequences with motif | 3099.5 |
| Percentage of Background Sequences with motif | 6.84% |
| Average Position of motif in Targets | 110.1 +/- 51.2bp |
| Average Position of motif in Background | 101.2 +/- 67.1bp |
| Strand Bias (log2 ratio + to - strand density) | 0.5 |
| Multiplicity (# of sites on avg that occur together) | 1.08 |
| Motif File: | file (matrix) reverse opposite |

### Similar de novo motifs found

|  |  |  |  |  |  |  |  |
| --- | --- | --- | --- | --- | --- | --- | --- |
| Rank | Match Score | Redundant Motif | P-value | log P-value | % of Targets | % of Background | Motif file |
| 1 | 0.868 | C G T A T C G A G C T A A T G C G T C A A C T G A C T G G T C A C T G A T G A C A G T C T A G C | 1e-23 | -54.274518 | 10.78% | 1.88% | motif file (matrix) |
| 2 | 0.893 | C G T A A G T C C G T A A T C G A C T G C G T A C G T A G T C A A G C T | 1e-13 | -30.174168 | 5.29% | 0.81% | motif file (matrix) |
